# The Interaction of Sex and Neuroplasticity in the Pathogenesis of Alzheimer's Disease

**DOI:** 10.1002/alz70856_106533

**Published:** 2026-01-09

**Authors:** Corey J Bolton, Panpan Zhang, Amalia Jo Peterson, Dandan Liu, Timothy J. Hohman, Kaj Blennow, Henrik Zetterberg, Angela L. Jefferson

**Affiliations:** ^1^ Vanderbilt Memory and Alzheimer's Center, Nashville, TN, USA; ^2^ Vanderbilt University Medical Center, Nashville, TN, USA; ^3^ Department of Biostatistics, Vanderbilt University Medical Center, Nashville, TN, USA; ^4^ Vanderbilt Memory & Alzheimer's Center, Vanderbilt University Medical Center, Nashville, TN, USA; ^5^ Department of Neurology, Vanderbilt University Medical Center, Nashville, TN, USA; ^6^ Vanderbilt Memory and Alzheimer's Center, Vanderbilt University Medical Center, Nashville, TN, USA; ^7^ Vanderbilt Memory and Alzheimer's Center, Vanderbilt University School of Medicine, Nashville, TN, USA; ^8^ Department of Psychiatry and Neurochemistry, Institute of Neuroscience and Physiology, The Sahlgrenska Academy, University of Gothenburg, Mölndal, Sweden; ^9^ Clinical Neurochemistry Laboratory, Sahlgrenska University Hospital, Mölndal, Sweden; ^10^ Clinical Neurochemistry Laboratory, Sahlgrenska University Hospital, Mölndal, Västra Götalands län, Sweden; ^11^ Department of Psychiatry and Neurochemistry, University of Gothenburg, Gothenburg, Sweden; ^12^ Hong Kong Center for Neurodegenerative Diseases, Hong Kong, China; ^13^ Wisconsin Alzheimer's Disease Research Center, School of Medicine and Public Health, University of Wisconsin‐Madison, Madison, WI, USA; ^14^ UK Dementia Research Institute at UCL, London, United Kingdom; ^15^ Department of Neurology, Vanderbilt Memory & Alzheimer's Center, Vanderbilt University Medical Center, Nashville, TN, USA

## Abstract

**Background:**

Women are at an increased risk of dementia due to Alzheimer's disease (AD) compared to men, a difference due in part to the role of female sex hormones. Estrogen, in particular, plays a key role in neuroplasticity. However, as women age and estrogen levels decline, high levels of neuroplasticity may be unsustainable. This study investigates the interaction of sex with a marker of neuroplasticity, growth‐associated protein‐43 (GAP‐43), on AD biomarkers and cognitive decline.

**Method:**

Vanderbilt Memory and Aging Project participants free of clinical dementia or stroke (*n* = 161, 72±6 years, 31% female) underwent fasting lumbar puncture and comprehensive neuropsychological assessment at study entry and serially over a mean 6.4‐year follow‐up period. Cerebrospinal fluid (CSF) levels of GAP‐43, b‐amyloid_1‐42_ (Ab_1‐42_), tau, and phosphorylated‐tau (*p*‐tau) were analyzed in batch. Linear regression models related baseline CSF GAP‐43 cross‐sectionally and longitudinally to CSF biomarkers and cognition adjusting for baseline age, sex, education, race/ethnicity, *apolipoprotein E* (*APOE*)‐e4 status, modified Framingham Stroke Risk Profile, and cognitive status. Follow‐up models assessed *GAP‐43 x sex* interactions on AD biomarkers and cognitive outcomes.

**Result:**

In cross‐sectional analyses, higher GAP‐43 was associated with higher levels of all CSF AD biomarkers (*p*‐values<0.0001), worse language performance (b=‐0.0005, *p* = 0.04) and worse visuospatial performance (b=‐0.0004, *p* = 0.005). GAP‐43 interacted with sex on CSF tau and *p*‐tau levels (*p*‐values<0.0001) such that associations were stronger in females compared to males. In longitudinal analyses, higher baseline GAP‐43 was associated with declining Ab42 levels (b=‐0.01, *p* <0.0001) and declining performance in tasks of language, executive function, and visuospatial abilities (*p*‐values<0.02), indicating greater AD pathology and declining cognition. GAP‐43 interacted with sex on longitudinal language, processing speed, executive functioning, and visuospatial performance trajectories (*p*‐values<0.05), such that significant associations were found in women (*p*‐values<0.02) but not men (*p*‐values>0.26).

**Conclusion:**

In this cohort of community‐dwelling older adults, we found higher baseline levels of GAP‐43, indicating increased neuroplasticity, are related to cross‐sectional increases in tau pathology, especially in women, and longitudinal decline in cognition, exclusively in women. Findings suggest that differential responses to neuroplasticity in aging could help explain long‐recognized sex differences in tau pathology and cognitive decline.

